# Activation of the SPHK/S1P signalling pathway is coupled to muscarinic receptor-dependent regulation of peripheral airways

**DOI:** 10.1186/1465-9921-6-48

**Published:** 2005-05-31

**Authors:** Melanie Pfaff, Norbert Powaga, Sibel Akinci, Werner Schütz, Yoshiko Banno, Silke Wiegand, Wolfgang Kummer, Jürgen Wess, Rainer Viktor Haberberger

**Affiliations:** 1 Institute for Anatomy and Cell Biology Justus-Liebig-University Giessen, Germany; 2 Department of Cell Signaling, Graduate School of Medicine, Gifu University, Gifu, Japan; 3 Laboratory of Bioorganic Chemistry, National Institute of Diabetes and Digestive Kidney Diseases, Bethesda, Maryland 20892, USA

## Abstract

**Background:**

In peripheral airways, acetylcholine induces contraction via activation of muscarinic M2-and M3-receptor subtypes (M_2_R and M_3_R). Cholinergic hypersensitivity is associated with chronic obstructive pulmonary disease and asthma, and therefore the identification of muscarinic signaling pathways are of great therapeutic interest. A pathway that has been shown to be activated via MR and to increase [Ca^2+^]_i _includes the activation of sphingosine kinases (SPHK) and the generation of the bioactive sphingolipid sphingosine 1-phosphate (S1P). Whether the SPHK/S1P signaling pathway is integrated in the muscarinic control of peripheral airways is not known.

**Methods:**

To address this issue, we studied precision cut lung slices derived from FVB and M_2_R-KO and M_3_R-KO mice.

**Results:**

In peripheral airways of FVB, wild-type, and MR-deficient mice, SPHK1 was mainly localized to smooth muscle. Muscarine induced a constriction in all investigated mouse strains which was reduced by inhibition of SPHK using D, L-threo-dihydrosphingosine (DHS) and N, N-dimethyl-sphingosine (DMS) but not by N-acetylsphingosine (N-AcS), a structurally related agent that does not affect SPHK function. The initial phase of constriction was nearly absent in peripheral airways of M_3_R-KO mice when SPHK was inhibited by DHS and DMS but was unaffected in M_2_R-KO mice. Quantitative RT-PCR revealed that the disruption of the M_2_R and M_3_R genes had no significant effect on the expression levels of the SPHK1-isoform in peripheral airways.

**Conclusion:**

These results demonstrate that the SPHK/S1P signaling pathway contributes to cholinergic constriction of murine peripheral airways. In addition, our data strongly suggest that SPHK is activated via the M_2_R. Given the important role of muscarinic mechanisms in pulmonary disease, these findings should be of considerable therapeutic relevance.

## Background

Acetylcholine (ACh), released from parasympathetic nerve fibres, leads to bronchoconstriction via stimulation of muscarinic acetylcholine receptors (MRs) and subsequent increase in intracellular calcium levels [Ca^2+^]_i_. The MR-family consists of five molecularly distinct subtypes (M_1–5_R) that are coupled to heterotrimeric G-proteins [1,2]. Activation of the M_2_R and M_4_R subtypes generally decreases intracellular cAMP levels, whereas stimulation of the M_1_R, M_3_R and M_5_R subtypes leads to the activation of phospholipase Cβ (PLCβ) with subsequent generation of the second messenger inositol 1, 4, 5-trisphosphate (IP_3_) and an increase in [Ca^2+^]_I _[1,2]. Asthmatic patients show hypersensitivity to MR agonists, and consequently antimuscarinic agents are commonly used in treatment of upper and lower airway diseases [3].

Several studies suggest that the PLC-IP_3_-signalling pathway is not solely responsible for changes in [Ca^2+^]_I_. For example, activation of the cyclic ADP-ribose pathway abolishes ACh-induced Ca^2+ ^oscillations in smooth muscle of porcine airways (White et al. 2003). Another alternative pathway that has been shown to increase [Ca^2+^]_i _involves the activation of sphingosine kinases (SPHK) and the subsequent generation of the bioactive sphingolipid sphingosine 1-phosphate (S1P) [4-6]. While S1P is well known as an important extracellular mediator of many biological pathways, including cell survival, angiogenesis and cell migration, recent studies indicate that S1P is also an intracellular second messenger which is coupled to changes in intracellular Ca^2+ ^levels [4-6]. In the airways, S1P has been shown to stimulate airway smooth muscle proliferation and cytokine release [7]. When applied to cultured human tracheal myocytes, S1P also increases [Ca^2+^]_i _and evokes contractile responses [7,8]. Moreover, muscarinic activation of M_2_R-and M_3_R-transfected HEK293 cells stimulates S1P synthesis [4,5]. In the present study we therefore tested the hypothesis that activation of the SPHK/S1P signalling pathway may contribute to MR-dependent regulation of peripheral airway diameter. For all studies we used airways from wild-type (wt) and M_2_R and M_3_R mutant mice that were ~200 μm in diameter. It is well known that smaller airways are mainly responsible for airway resistance [9,10]. Moreover previous studies clearly demonstrated differences between larger and smaller airways concerning functional responses, receptor expression or ion conductance [11].

In murine tissues, S1P is synthesized after activation of two SPHK isoforms, SPHK1 and SPHK2. SPHK1 is highly expressed in adult lung [12-14], whereas the SPHK2 isoform shows much lower expression in lung tissue [15]. SPHK2 contains a nuclear localization sequence and has recently been identified as a nuclear protein capable of inhibiting DNA synthesis, whereas SPHK1 is mainly localized in the cytosol [14,16]. In the present study we therefore focused exclusively on the expression and potential functional role of the SPHK1 isoform in MR-mediated airway constriction.

The expression of the SPHK1 isoform was investigated by means of quantitative RT-PCR and immunohistochemistry using precision cut lung slices (PCLS) of murine lungs [17]. Using murine PCLS we also measured the constriction of small intraparenchymal airways in response to MR activation. Coupling of MRs to SPHK-activation was investigated by blocking SPHK with D, L-threo-dihydrosphingosine (DHS) or N, N-dimethyl-sphingosine (DMS, [18]). Moreover, the role of MR-dependent intracellular Ca^2+ ^release on airway diameter was studied by inhibiting Ca^2+^-influx by La^3+ ^and SKF 96365. Recent evidence indicates that MR agonists induce bronchoconstriction by activating a mixture of M_2_R and M_3_R subtypes, present on smooth muscle cells of extra-and intraparenchymal airways [19,20]. To study the potential roles of the M_2_R and M_3_R subtypes in SPHK1-dependent peripheral airway responses, we therefore also carried out functional studies with PCLS prepared from M_2_R-and M_3_R-deficient mice (M_2_R-KO, M_3_R-KO) and their corresponding wild type controls (M_2_R-wt, M_3_R-wt) [21,22].

## Methods

### Animals

Lungs were taken from 8-12 wk old FVB mice (Harlan-Winkelmann, Borchen, Germany), mice deficient in M_2_R or M_3_R (M_2_R-KO, M_3_R-KO) and their corresponding wild-type strains (M_2_R-wt, M_3_R-wt). The generation of M_2_R-KO-and M_3_R-KO mice has been described previously [21,22]. The M_2_R-KO mice and the M_2_R-wt mice had the following genetic background: 129J1 (50%) × CF1 (50%). The M_3_R-KO mice and the corresponding wild-type mice had the following genetic background: 129SvEv (50%) × CF1 (50%). The animals were killed by cervical dislocation. The mice were kept under specific pathogen free conditions until the experiments.

### Quantitative RT-PCR

Real-time quantitative PCR (iCycler, Bio-Rad, München, Germany) was used to quantify levels of SPHK1 mRNA in PCLS of FVB, M_2_R-KO, M_3_R-KO, M_2_R-wt, and M_3_R-wt mice. Lung slices were transferred into lysis buffer (Qiagen, Heiden, Germany) and homogenized using a mixer mill with a frequency of 300 Hz (Qiagen). Total RNA was isolated according to the protocol recommended by the manufacturer (Rneasy kit, Qiagen). Contaminating DNA was removed using DNase (1 U/μg total RNA, Gibco-BRL, Life Technologies, Karlsruhe, Germany) in the presence of 20 mM Tris-HCl (pH 8.4), 2 mM MgCl_2_, 50 mM KCl for 15 min at 25°C. Equal amounts of RNA were reverse transcribed in the presence of 3 mM MgCl_2_, 75 mM KCl, 50 mM Tris-HCl (pH 8.3), 10 mM dithiothreitol, 0.5 mM dNTPs (Gibco-BRL) and 25 μg oligo (dT) (MWG Biotech, Ebersberg, Germany), with 200 U of Superscript RNase H^- ^Reverse transcriptase (Gibco-BRL) for 50 min at 42°C. Gene specific PCR primers for mouse SPHK1 and β_2_-microglobulin (SPHK1, gi:22094104, fw TCCAGAAACCCCTGTGTAGC, rev GCTCCCTAGGGCCAGTAAAC product size 188 bp, β2-microglobulin gi:12861272 fw ATGGGAAGCCGAACATACTG, rev CAGTCTCAGTGGGGGTGAAT, product size 176 bp) were designed using Primer Express™ software (Applied Biosystems, Foster City, USA). All PCR-reactions were prepared in triplicate from four to eight animals using a ready-to-use kit according to the manufacturers protocol (QuantiTect™ SYBR Green PCR Kit, Qiagen). Primers specific for β-microglobulin were used for standardisation. The data were normalised by subtracting the threshold cycle (CT) levels between SPHK1 and β_2_-microglobulin. In each independent experiment qRT-PCR reactions were performed in triplicate.

### Double-labelling immunofluorescence

Slices (220 μm thick) prepared for videomorphometry were fixed for 20 min in ice-cold acetone and washed repeatedly in 0.1 M phosphate buffer. Sections were covered for 1 h with blocking medium (50 % normal porcine serum in PBS) followed by overnight incubation with an antiserum directed against the SPHK1 isoform (1:400, [23]) in combination with a monoclonal FITC-labelled anti-α-smooth muscle actin antibody (1:500, clone 1A4, Sigma, Deisenhofen, Germany). The sections were then washed in PBS and covered for 1 h with Cy3-conjugated donkey anti-rabbit Ig antiserum (1:3000, Dianova, Hamburg, Germany). After incubation with the secondary antibody, the slides were washed in PBS and coverslipped in carbonate-buffered glycerol at pH 8.6. Omission of primary antisera or preabsorption of the SPHK1a antiserum with the corresponding synthetic peptide (20–100 μg antigen/ml diluted antiserum) abolished immunolabelling. The slides were evaluated by sequential confocal laser scanning microscopy (TCPSP, Leica, Bensheim, Germany) using the appropriate laser for Cy3 (excitation 543 nm) and FITC (excitation 488 nm).

### Videomorphometry

PCLS were prepared using a slightly modified version of the protocol described by Martin et al. [24]. Briefly, the mice were killed by cervical dislocation and the lungs were perfused via the right ventricle with 37°C Krebs-Ringer buffer containing heparin (1000 I.U.), penicillin/streptomycin (1 %) and sodium nitroprusside (0.075 μM). The airways were filled via the cannulated trachea with agarose (low melting point agarose, 1.6 % in Krebs-Ringer buffer, Sigma, Deisenhofen, Germany). Subsequently, the lungs and heart were removed *en bloc*, placed in ice-cold HEPES-Ringer buffer and cut in 200–250 μm thick slices using a vibratome (VT1000S, Leica). Subsequently, the precision cut lung slices (PCLS) were incubated in minimal essential medium (MEM) at 37°C for 4–7 h. Experiments were performed in a lung slice superfusion chamber (Hugo Sachs Elektronik, March, Germany) mounted on an inverted microscope (Leica). Images were recorded using a CCD-camera (Stemmer Imaging, Puchheim, Germany) and analyzed using the Optimas 6.5 image analysis software (Stemmer). The slices were fixed in the chamber with nylon strings that were connected to a platinum ring. Viable airways of about 200 μm in diameter were examined and incubated in the slide chamber for 5 min in HEPES-Ringer buffer until the first image was acquired. The area of the airway lumen at the beginning of the experiment was defined as 100 % and bronchoconstriction or dilatation were expressed as relative decrease or increase of this area. Data from FVB mice and wt-strains were used only from those experiments where the reduction of luminal area in response to 10^-6 ^M muscarine reached at least 25 %. Muscarine, [propoxy]-ethyl-1H-imidazole] (SKF96365), lanthanum chloride and N-acetylspingosine (N-AcS) were purchased from Sigma. D, L-threo-dihydrosphingosine (DHS) and N,N-dimethyl-sphingosine (DMS) were purchased from Biomol (Hamburg, Germany).

### Statistical analysis

Data are presented as means ± standard error of the mean (SEM) of 5–10 slices obtained from five to nine animals. Matched pairs were evaluated by Wilcoxon's rank sum test. In the case of more than 2 non-matched groups, Mann-Whitney U-test for comparison between two groups was conducted only when statistically significant differences were reached by the global Kruskal-Wallis test that was performed first. Differences were considered as statistically significant when p < 0.05.

## Results

The goal of the present study was to determine the potential involvement of SPHK1 activation in the MR-mediated constriction of small peripheral airways. To be able to measure the diameter of small murine airways, we used videomicroscopy of viable precision-cut lung slices (PCLS; [24,20]). Specifically, we analyzed the effects of SPHK1 blockade in FVB and M_2_R and M_3_R single-knockout mice (M_2_R-KO and M_3_R-KO mice, respectively) as well as the corresponding wild-type control animals (M_2_R-wt and M_3_R-wt mice, respectively).

### qRT-PCR

**Figure 1:**
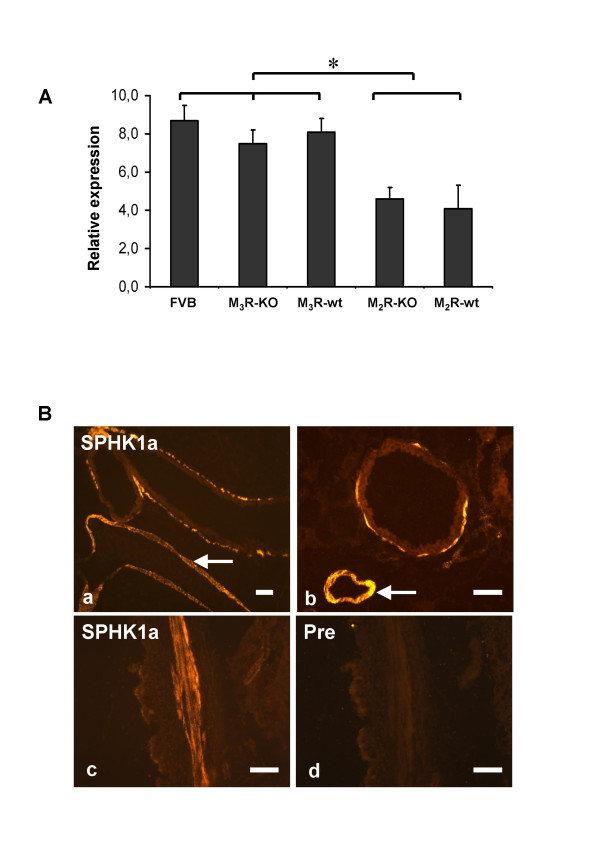
A) Quantitative RT-PCR analysis of SPHK1 expression in PCLS from different mouse strains. The relative expression of SPHK1-mRNA in relation to the house-keeping gene β_2 _microglobulin is shown. Data are given as means ± S.E.M. of four independent experiments each carried out in triplicate. B) Indirect immunofluorescence studies examining SPHK1 expression in PCLS of FVB-mice. Immunoreactivity for SPHK1 was present in the wall of larger (a) and smaller (b) airways and in the media of pulmonary vessels (arrows in a, b). Consecutive sections (c, d) showing that preabsorption of the SPHK1 antiserum abolished immunolabelling (Pre, d). Bars = 50 μm.

We used qRT-PCR analysis to quantitate and compare SPHK1-mRNA levels between FVB mice, M_2_R-and M_3_R-deficient mice (M_2_R-KO, M_3_R-KO), and the corresponding wt control mice (M_2_R-wt, M_3_R-wt). Expression of β_2_-microglobulin served as an internal control. We found that inactivation of the M_2_R and M_3_R genes had no significant effect on the relative expression levels of SPHK1 compared to their corresponding wt controls (Mann-Whitney U-test, Fig. 1A). This analysis also showed that SPHK1 expression was significantly lower in M_2_R-wt and M_2_R-KO mice, as compared to FVB, M_3_R-wt, and M_3_R-KO-mice (p < 0.05, Mann-Whitney U-test, Fig. 1A), perhaps due to the fact that the M_2_R-KO/wt mice have a genetic background that is somewhat different from that of the other mice used (for details see "Materials and Methods").

### Immunohistochemistry

**Figure 2:**
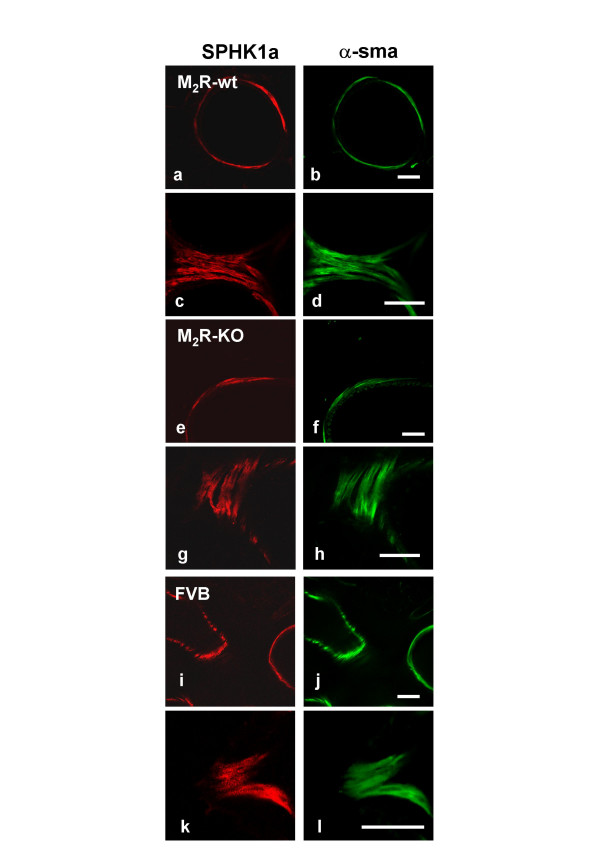
Expression of SPHK1 in PCLS from different mouse strains studied via confocal laser scanning microscopy. Representative confocal laser scanning micrographs of double labeling immunohistochemistry demonstrates the restriction of SPHK1-immunoreactivity to smooth muscle cells, identified by immunoreactivity for the marker protein α-smooth muscle actin (α-sma). SPHK1a-immunoreactivity was present in peripheral airway smooth muscle of knock-out animals (M_2_R-KO, a-d) and wild-type (M_2_R-wt, e-h, FVB, i-l). Granular immunoreactivity could be detected in the cytoplasm and in the membrane of smooth muscle cells. Bars = 50 μm

We used single-and double-labeling immunohistochemistry to examine the distribution of SPHK1 in murine peripheral lung. Strong SPHK1-immunoreactivity was detected in the smooth muscle cells of intraparenchymal bronchi (Figs. 1B, 2) and pulmonary arteries (Fig. 1B) and veins as confirmed by double-staining of a marker of smooth muscle cells, α-smooth muscle actin. Preabsorption of the SPHK1 antiserum abolished immunolabelling (Fig. 1B). Immunoreactivity for SPHK1 was absent in bronchial epithelium (Figs. 1B, 2). SPHK1 showed a granular cytoplasmatic localisation but was also found in smooth muscle membranes (Fig. 2). The pattern of SPHK1 immunoreactivity was very similar in PCLS of FVB, M_2_R-KO/wt, and M_3_R-KO/wt mice..

### Videomorphometry

**Figure 3:**
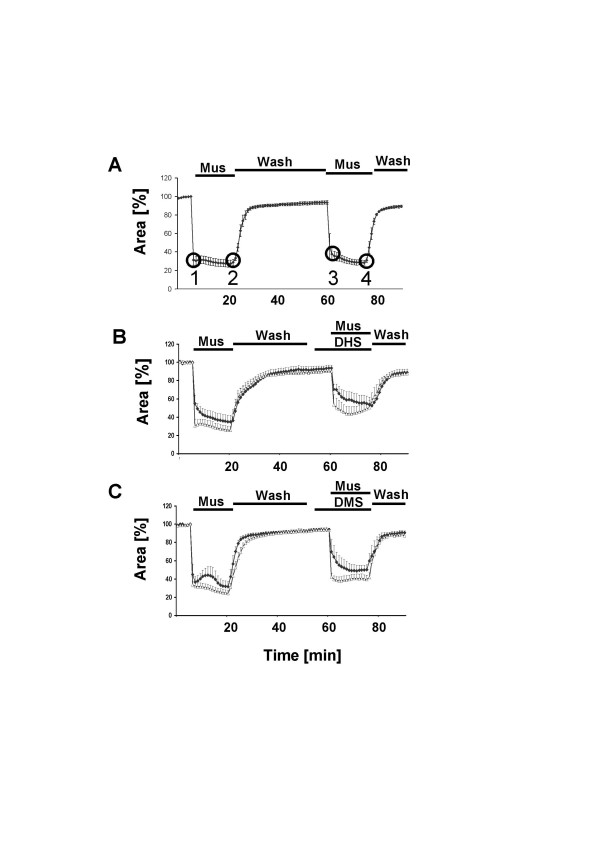
Muscarine-induced reductions in luminal area of peripheral bronchi from FVB mice. (A) Luminal area of peripheral bronchi after application of 10^-6 ^M muscarine (Mus) as recorded by videomorphometry. Data (means ± S.E.M) were expressed as % luminal area. Bronchi from FVB mice (4 slices from 6 lungs) responded to 10^-6 ^M muscarine until wash out (Wash). Time points 1–4 were chosen as indicators for initial and sustained constriction. 1 = luminal airway area 1 min after muscarine application, 2 = luminal airway area 15 min after muscarine application, 3 = luminal airway area 1 min after repeated muscarine application, 4 = luminal airway area 15 min after repeated muscarine application. Effect of DHS (B) and DMS (C) on muscarine-mediated reductions in luminal area of peripheral bronchi from FVB mice. The luminal area of peripheral bronchi (expressed in %) was recorded by videomorphometry after application of 10^-6 ^M muscarine (Mus) alone and after application of 10^-6 ^M muscarine together with (B) 10^-6 ^M DHS (diamonds, 7 slices from 4 lungs) and 10^-10 ^M DHS (triangles, 8 slices from 4 lungs) or (C) 10^-6 ^M DMS (diamonds, 9 slices from 7 lungs) and 10^-10 ^M DMS (triangles, 6 slices from 4 lungs). Bronchoconstriction responses were significantly reduced in the presence of DHS and DMS. The inhibition was more pronounced following coadministration of DMS. Data are given as means ± S.E.M.

*FVB mice *In PCLS preparations from FVB mice, the luminal area of peripheral bronchi rapidly decreased within the first 2 minutes after application of 10^-6 ^M muscarine, followed by a sustained decrease in presence of the agonist (Tab. 1, Fig. 3). Application of the SPHK inhibitors DMS and DHS alone for ten minutes prior to coadministration of 10^-6 ^M muscarine had no significant effect on bronchial luminal diameter (Figs. 3A, B, 5A–D). Coadministration of 10^-6 ^M muscarine with DHS (10^-4 ^M-10^-10 ^M) significantly reduced both the initial and the sustained phase of the muscarine induced bronchoconstriction (Tab. 1, Fig. 3B). Similarly, coadministration of 10^-6 ^M muscarine with 10^-6^-10^-10 ^M DMS reduced both phases of the muscarine response. However 10^-4 ^M DHS had no effect (Tab. 1). Coadministration of 10^-6 ^M muscarine with the structurally related sphingolipid N-acetylsphingosine (10^-6^-10^-10 ^M) which has no effect on SPHK1 function [5] did not alter the muscarine induced constriction (Tab. 1).

**Table 1:** Effects of N-AcS, DHS and DMS on muscarine-induced reductions in luminal airway area in FVB mice. The number of experiments (lungs/slices), mean airway diameter in μm, and the luminal airway area determined 1 min (1) and 15 min (2) after stimulation with muscarine (Mus) and 1 min (3) and 15 min (4) after repeated stimulation with muscarine or after repeated stimulation with muscarine in combination with N-AcS, DHS or DMS are shown. In all experiments, the muscarine concentration was 10^-6 ^M. Data are given as means ± S.E.M. Matched pairs (1 vs. 3; 2 vs. 4) were evaluated by Wilcoxon's rank sum test. Differences were considered as statistically significant when p < 0.05 (n.s., not significant).

Lungs /slices	Diameter [μm]	Area [%] 1	Area [%] 2	Area [%] 3	Area [%] 4	p (1 vs. 3)	p (2 vs. 4)
		Mus		Mus		n.s.	n.s.
4/4	206 ± 7	31 ± 6	38 ± 4	38 ± 7	28 ± 3		
		Mus		Mus/N-AcS 10^-6 ^M		n.s.	n.s.
6/5	182 ± 7	47 ± 8	42 ± 8	50 ± 8	42 ± 9		
		Mus		Mus/N-AcS 10^-8 ^M		n.s.	n.s.
5/5	218 ± 5	36 ± 1	28 ± 5	38 ± 6	26 ± 6		
		Mus		Mus/N-AcS 10^-10 ^M		n.s.	n.s.
4/4	212 ± 22	21 ± 8	16 ± 5	26 ± 6	30 ± 5		
		Mus		Mus/DHS 10^-4 ^M		n.s.	n.s.
4/6	194 ± 9	42 ± 5	37 ± 7	61 ± 8	45 ± 6		
		Mus		Mus/DHS 10^-6 ^M		p < 0.05	p < 0.05
7/7	197 ± 14	45 ± 11	31 ± 7	70 ± 13	50 ± 5		
		Mus		Mus/DHS 10^-8 ^M		p < 0.05	p < 0.05
7/9	181 ± 6	39 ± 7	29 ± 6	50 ± 9	47 ± 7		
		Mus		Mus/DHS 10^-10 ^M		p < 0.05	p < 0.05
4/8	198 ± 6	34 ± 3	24 ± 3	42 ± 4	40 ± 4		
		Mus		Mus/DMS 10^-4 ^M		p < 0.05	p < 0.05
4/7	180 ± 7	30 ± 7	25 ± 6	45 ± 10	41 ± 7		
		Mus		Mus/DMS 10^-6 ^M		p < 0.05	p < 0.05
4/7	201 ± 11	48 ± 7	35 ± 8	70 ± 6	54 ± 9		
		Mus		Mus/DMS 10^-8 ^M		p < 0.05	p < 0.05
4/7	206 ± 11	31 ± 5	25 ± 4	54 ± 8	50 ± 5		
		Mus		Mus/DMS 10^-10 ^M		p < 0.05	p < 0.05
4/6	196 ± 15	43 ± 6	23 ± 4	71 ± 11	52 ± 7		

**Figure 4:**
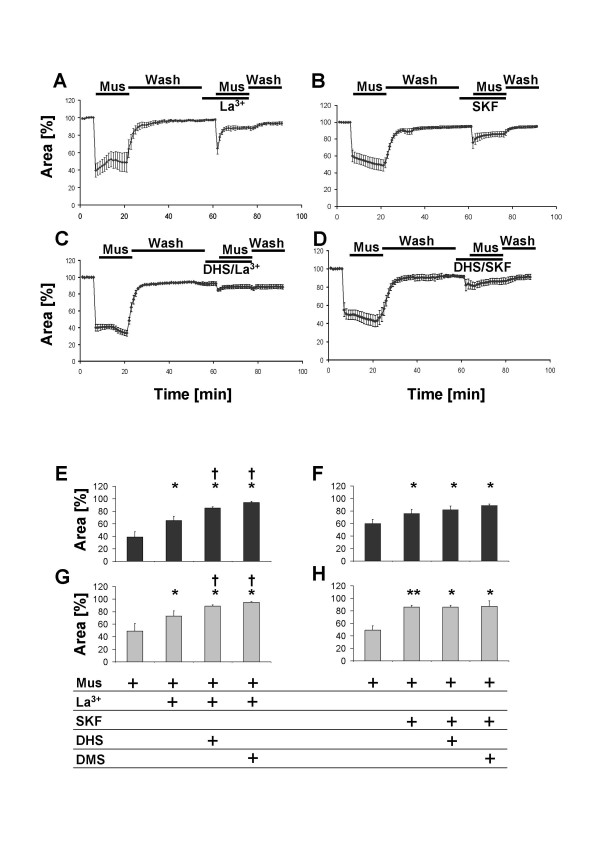
Effect of various treatments on muscarine-mediated reductions in luminal area of peripheral bronchi from FVB mice. The luminal area of peripheral bronchi (expressed in %) was recorded by videomorphometry after application of 10^-6 ^M muscarine alone or after application of 10^-6 ^M muscarine in the presence of (A) 10^-6 ^M La^3+ ^(7 slices from 4 lungs), (B) SKF 96365 (SKF 10^-5 ^M, 9 slices from 4 lungs), (C) 10^-6 ^M La^3+ ^in combination with 10^-6 ^M DHS (7 slices from 4 lungs), or (D) 10^-5 ^M SKF 96365 in combination with 10^-6 ^M DHS (8 slices from 4 lungs). Data are given as means ± S.E.M.E-F) Summary of effects of various treatments on muscarine-mediated reductions in luminal area of peripheral bronchi from FVB mice. Luminal area (expressed in %) determined 1 min (initial contraction, E) and 15 min (sustained contraction, G) after application of 10^-6 ^M muscarine alone or of muscarine in combination with either La^3+ ^(both 10^-6 ^M) or La^3+ ^in combination with DHS or DMS (both 10^-8 ^M). Luminal area (expressed in %) determined 1 min (initial contraction, F) and 15 min (sustained contraction, H) after application of muscarine alone or of muscarine in combination with either SKF 96365 (10^-5 ^M) or SKF 96365 in combination with 10^-8 ^M DHS or DMS. Asterisks indicate p < 0.05 for the comparison with application of muscarine alone (E-F). Daggers indicate p < 0.05 for the comparison with application of muscarine in combination with La^3+ ^(E, G). Data are given as means ± S.E.M.

Inhibition of Ca^2+ ^entry by La^3+ ^(10^-6 ^M) considerably reduced the initial and strongly inhibited the sustained constriction of peripheral airways following the administration of 10^-6 ^M muscarine (Tab. 2, Fig. 4A, E, G). Combination of 10^-8 ^M DHS or 10^-8 ^M DMS with La^3+ ^(10^-6 ^M) almost completely abolished the initial and the sustained phase of the muscarine-dependent bronchoconstriction (Tab. 2, Fig. 4C, E, G). The combination of DHS or DMS with La^3+ ^further significantly reduced both phases compared to La^3+ ^alone (Wilcoxon's rank sum test p < 0.05)

**Table 2:** Effects of SKF96365 and La^3+ ^alone and in combination with DHS or DMS on the muscarine-induced reductions in luminal airway area in FVB mice Effects of the Ca^2+^-entry inhibitors La^3+ ^(10^-6 ^M) and SKF 96365 (10^-5 ^M) alone and in combination with DHS or DMS (both 10^-6 ^M) on the muscarine induced reduction in luminal airway area (expressed in %). The number of experiments (lungs/slices), mean airway diameter in μm, and the luminal airway area determined 1 min (1) and 15 min (2) after stimulation with muscarine and 1 min (3) and 15 min (4) and after stimulation with muscarine (Mus) in combination with La^3+ ^(Mus/La^3+^) or SKF96365 (Mus/SKF), or after stimulation with muscarine and La^3+ ^or SKF96365 in combination with DHS or DMS are shown. In all experiments, the muscarine concentration was 10^-6 ^M. Data are given as means ± S.E.M. Matched pairs (1 vs. 3; 2 vs. 4) were evaluated by Wilcoxon's rank sum test. Differences were considered as statistically significant when p < 0.05.

Lungs /slices	Diameter [μm]	Area [%] 1	Area [%] 2	Area [%] 3	Area [%] 4	p (1 vs. 3)	p (2 vs. 4)
4/9	212 ± 3	Mus		Mus/SKF		p < 0.05	p < 0.05
		60 ± 7	49 ± 7	76 ± 7	86 ± 3		
4/8	193 ± 12	Mus		Mus/SKF/DHS		p < 0.05	p < 0.05
		51 ± 5	43 ± 6	82 ± 6	86 ± 3		
5/6	207 ± 11	Mus		Mus/SKF/DMS		p < 0.05	p < 0.05
		65 ± 3	53 ± 6	88 ± 2	85 ± 8		
4/7	180 ± 8	Mus		Mus/La^3+^		p < 0.05	p < 0.05
		39 ± 8	49 ± 12	65 ± 7	89 ± 2		
4/7	211 ± 12	Mus		Mus/La^3+^/DHS		p < 0.05	p < 0.05
		40 ± 4	33 ± 3	85 ± 2	89 ± 2		
4/5	210 ± 15	Mus		Mus/La^3+^/DMS		p < 0.05	p < 0.05
		45 ± 9	38 ± 9	94 ± 2	95 ± 1		

Blockade of Ca^2+^-entry by SKF96365 (10^-5 ^M), which inhibits G-protein activated and voltage gated Ca^2+^-channels, greatly reduced both the initial and the sustained phase of muscarine-induced bronchoconstriction (Tab. 3, Fig. 4B, F, H). Combination of SKF96365 (10^-5 ^M) with 10^-8 ^M DMS or 10^-8 ^M DHS exerted no further reduction of luminal airway area (Wilcoxon's rank sum test, Tab. 2, Fig. 4F, H).

**Figure 5:**
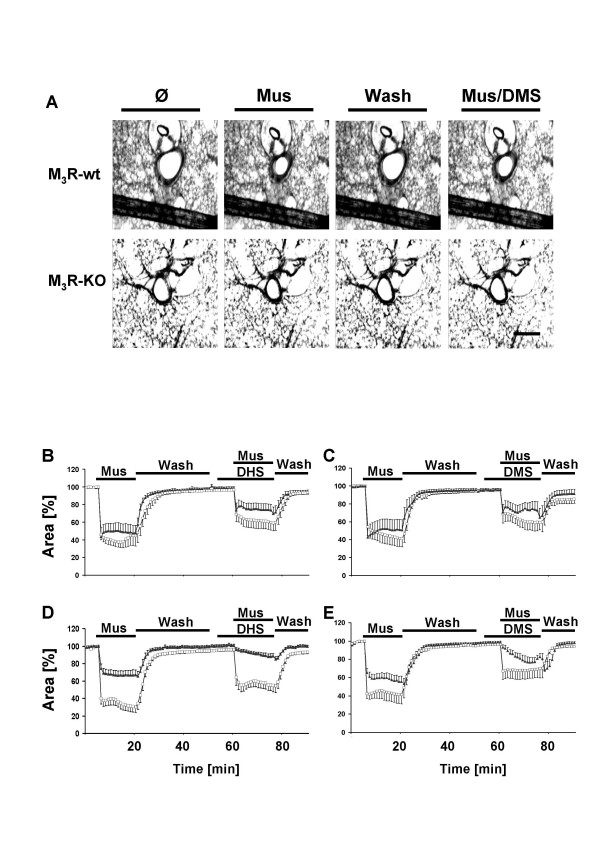
Muscarine-mediated changes in luminal area of peripheral bronchi from wild-type and MR deficient mice. Changes in luminal area of mouse peripheral airways were recorded by videomorphometry after cumulative application of different concentrations of muscarine. (A) Videomorphometric images of precision cut lung slices before (∅), 1 min after stimulation with muscarine (10^-6 ^M), after 15 min wash out (Wash) and 1 min after stimulation with muscarine in presence of DMS (10^-8 ^M). The bronchi from wild-type (M_3_R-wt) and M_3_R-KO mice constricted in response to 10^-6 ^M muscarine. In M_3_R-wt mice, the constriction was slightly reduced in presence of DMS but abolished in M_3_R-KO mice. Bar = 200 μm. (B-D) Effect of DHS and DMS on muscarine-mediated reductions in luminal area of murine peripheral bronchi in the absence of the M_2_R or M_3_R subtypes. (B-C) Luminal area of peripheral airways from M_2_R-KO (triangles) and M_2_R-wt mice (squares)(expressed in % in response to application of 10^-6 ^M muscarine (Mus) alone or after application of muscarine (10^-6 ^M) in combination with DHS (B) or DMS (C) (both 10^-8 ^M). Both DHS and DMS significantly inhibited the muscarine-induced constriction in airways of both M_2_R-KO and M_2_R-wt mice. (D, E) Luminal area of peripheral airways (expressed in %) from M_3_R-KO (triangles) and M_3_R-wt (squares) mice in response to application of 10^-6 ^M muscarine alone or after application of muscarine (10^-6 ^M) in combination with DHS (D) or DMS (E) (both 10^-8 ^M). Both DHS and DMS significantly inhibited the muscarine-induced constriction in airways of both M_3_R-KO and M_3_R-wt mice. Data are given as means ± S.E.M.

*M*_*2*_*R-KO/wt mice *Muscarine (10^-6 ^M) stimulation of PCLS preparations from M_2_R-KO mice resulted in an initial constriction response that was followed by a slight relaxation (4–8 % relaxation of luminal airway area within 3–15 min, 28 slices/27 lungs, Tab. 3, Fig. 5A, B). In contrast, in PCLS preparations from the corresponding wt mice (M_2_R-wt), the initial bronchoconstriction was followed by a sustained constriction response. In PCLS of M_2_R-KO and M_2_R-wt mice, DHS and DMS (10^-8 ^M each) significantly inhibited the initial and sustained phase of muscarine induced constriction (Tab. 3, Fig. 5A, B). The initial constriction was inhibited by about 54 % (luminal area of M_2_R-wt: 48 ± 11.5 %; M_2_R-KO: 61.5 ± 9.5 %, Tab. 3, Fig. 5A, B) and the sustained phase by about 50 % (luminal area of M_2_R-wt: 34 ± 12 %; M_2_R-KO: 67.5 ± 15 %, Tab. 3, Fig. 5A, B). The degree of inhibition of the initial phase was comparable between M_2_R-wt and M_2_R-KO mice.

**Table 3:** Effects of DHS and DMS on muscarine-induced reductions in luminal airway area in PCLS from M_2 _R-KO and theircorresponding wild-type mice. The number of experiments (lungs/slices), mean airway diameter in μm, and the muscarine-induced constriction in luminal airway area (expressed in %) determined after 1 min (1) and 15 min (2) after stimulation with muscarine and 1 min (3) and 15 min (4) after stimulation with muscarine in combination with DHS or DMS (both 10^-6 ^M or 10^-8 ^M) are shown. In all experiments, the muscarine concentration was 10^-6 ^M. The initial phase (1 vs 3) and the sustained phase (2 vs 4) of constriction were reduced in the presence of DHS or DMS. Data are given as means ± S.E.M.

M_2_R-wt Lungs /slices	Diameter [μm]	Area [%] 1	Area [%] 2	Area [%] 3	Area [%] 4	p (1 vs. 3)	p (2 vs. 4)
6/7	208 ± 9	Mus		Mus/DHS 10^-6 ^M		p ≤ 0.05	p ≤ 0.05
		35 ± 9	32 ± 9	77 ± 11	52 ± 11		
7/8	213 ± 8	Mus		Mus/DHS 10^-8 ^M		n.s. p = 0.078	p ≤ 0.05
		46 ± 6	45 ± 12	61 ± 9	60 ± 9		
5/5	202 ± 17	Mus		Mus/DMS 10^-6 ^M		p ≤ 0.05	p ≤ 0.05
		40 ± 10	42 ± 9	72 ± 9	66 ± 10		
7/7	217 ± 8	Mus		Mus/DMS 10^-8 ^M		p ≤ 0.05	p ≤ 0.05
		54 ± 10	41 ± 9	69 ± 10	60 ± 10		
M_2_R-KO							
6/6	210 ± 8	Mus		Mus/DHS 10^-6 ^M		p ≤ 0.05	p ≤ 0.05
		50 ± 11	54 ± 11	79 ± 7	82 ± 7		
7/7	194 ± 9	Mus		Mus/DHS 10^-8 ^M		p ≤ 0.05	p ≤ 0.05
		44 ± 10	48 ± 8	74 ± 8	74 ± 7		
7/8	218 ± 8	Mus		Mus/DMS 10^-6 ^M		p ≤ 0.05	p ≤ 0.05
		56 ± 8	62 ± 7	83 ± 6	79 ± 6		
7/7	189 ± 9	Mus		Mus/DMS 10^-8 ^M		p ≤ 0.05	p ≤ 0.05
		43 ± 13	51 ± 11	71 ± 9	73 ± 9		

*M*_*3*_*R-KO/wt mice *As in all other experiments, the area of the airway lumen at the beginning of the experiment was defined as 100 %. Application of 10^-6 ^M muscarine constricted peripheral airways in wt-mice by about 66 % (28 slices/25 lungs), whereas in M_3_R-KO mice the constriction was reduced by about 57 % (29 slices/28 lungs, Tab. 4 Figs. 5C, D, 6). In wt-bronchi, inhibition of SPHK by DHS or DMS (10^-8 ^M each) significantly reduced the initial phase of constriction by about 50 % (58 ± 12 %, Tab. 4, Figs. 5C, D, 6). Strikingly, treatment of PCLS from M_3_R-KO mice with DHS (10^-8 ^M) or DMS (10^-6 ^and 10^-8 ^M) almost completely abolished the initial constriction response (94 ± 4 %, Tab. 4, Figs. 5, 6). Inhibition of SPHK by DHS (10^-8 ^M) or DMS (10^-6^and 10^-8 ^M) significantly reduced the sustained phase of constriction to a comparable degree in M_3_R-wt and M_3_R-KO mice (Tab. 4, Figs. 5C, D, 6).

**Table 4:** Effects of DHS and DMS on muscarine-induced reductions in luminal airway area in PCLS from M_3 _R-KO and their corresponding wild-type mice. The number of experiments (lungs/slices), mean airway diameter in μm, and the muscarine-induced constriction in luminal airway area (expressed in %) determined after 1 min (1) and 15 min (2) after stimulation with muscarine and 1 min (3) and 15 min (4) after stimulation with muscarine in combination with DHS or DMS (both 10^-6 ^M or 10^-8 ^M) are shown. In all experiments, the muscarine concentration was 10^-6 ^M. The initial phase (1 vs 3) and the sustained phase (2 vs 4) of constriction were reduced in presence of DHS or DMS. Data are given as means ± S.E.M.

M_3_R-wt Lungs /slices	Diameter [μm]	Area [%] 1	Area [%] 2	Area [%] 3	Area [%] 4	p (1 vs. 3)	p (2 vs. 4)
6/6	221 ± 10	Mus		Mus/DHS 10^-6 ^M		n.s.	p ≤ 0.05
		53 ± 7	47 ± 8	75 ± 7	69 ± 11		
8/9	204 ± 10	Mus		Mus/DHS 10^-8 ^M		p ≤ 0.05	p ≤ 0.01
		40 ± 6	30 ± 6	64 ± 9	55 ± 6		
6/7	203 ± 6	Mus		Mus/DMS 10^-6 ^M		p ≤ 0.05	p ≤ 0.05
		41 ± 10	37 ± 8	64 ± 10	56 ± 8		
5/6	197 ± 8	Mus		Mus/DMS 10^-8 ^M		p ≤ 0.05	p ≤ 0.05
		42 ± 5	41 ± 10	71 ± 7	69 ± 9		
M_3_R-KO							
4/4	205 ± 18	Mus		Mus/DHS 10^-6 ^M		n.s.	n.s.
		69 ± 2	51 ± 4	77 ± 8	62 ± 12		
8/9	188 ± 9	Mus		Mus/DHS 10^-8 ^M		p ≤ 0.05	p ≤ 0.05
		75 ± 6	67 ± 6	96 ± 2	88 ± 4		
7/7	202 ± 12	Mus		Mus/DMS 10^-6 ^M		p ≤ 0.05	p ≤ 0.05
		75 ± 4	67 ± 6	97 ± 1	82 ± 4		
9/9	196 ± 7	Mus		Mus/DMS 10^-8 ^M		p ≤ 0.01	p ≤ 0.05
		65 ± 5	56 ± 6	93 ± 2	81 ± 3		

## Discussion

MR signaling pathways play a key role in the regulation of airway resistance which is determined largely by the diameter of smaller, intrapulmonary airways [25]. A better understanding of the different pathways underlying MR activation in the intrapulmonary airways is of considerable clinical relevance. In the present study, we examined the hypothesis that S1P might be involved in the muscarinic control of peripheral airways. To address this question, we used the PCLS model which has been shown to maintain the integrity of all components of the peripheral lung including viable peripheral airways [20].

Using double labelling immunohistochemistry we demonstrated for the first time that the SPHK1 protein is highly expressed in the cytosol of murine airway smooth muscle cells with virtually no immunoreactivity in non-smooth muscle cells. On the other hand, human and murine lung tissue showed a high expression and activity of SPHK which was not restricted to smooth muscle alone [13,14]. The virtual restriction of SPHK1-immununoreactivity to smooth muscle could be due to the presence of SPHK isoforms other than SPHK1 in murine lung [14]. At the subcellular level, SPHK1 showed a cytoplasmic localisation but was also found in airway smooth muscle membranes, in agreement with functional and immunoprecipitation studies of mouse lung membranes [14].

In the present study, the use of membrane-permeable SPHK inhibitors, DHS and DMS [18], convincingly demonstrated that MR-mediated constriction of peripheral airways involves the activation of SPHK. In PCLS preparations from wt-mice, both SPHK inhibitors significantly reduced the fast initial constriction response (induced by 10^-6 ^M muscarine) which is mainly dependent on Ca^2+^-release from intracellular stores [26]. This effect was observed with the lowest concentrations of the inhibitors but was absent when DHS and DMS were given alone for ten minutes prior to the application of DHS and DMS in combination with muscarine. This inhibition of muscarine induced bronchoconstriction was also absent when we used N-acetylsphingosine (N-AcS), an agent that is structurally related to DHS and DMS but that does not affect SPHK function [5]. These data suggest that DHS and DMS did not exert their inhibitory effects through nonspecific actions. Accordingly, the muscarine-induced initial bronchoconstriction was partly inhibited but persisted in presence of the blockers of Ca^2+^-influx, La^3+ ^or SKF 96365 [27,28], but was almost abolished when La^3+ ^was applied in combination with DHS or DMS. The sustained phase of muscarine-induced bronchoconstriction was also significantly inhibited by DHS and DMS in FVB mice. Interestingly, the responses that were inhibited by DHS/DMS were SKF96365-but not La^3+^-sensitive, since DHS/DMS significantly increased the La^3+^-mediated inhibition of the muscarine-mediated constriction, but had no effect on the SKF96365-mediated inhibition of the constriction.

MR-mediated constriction of peripheral airways has been shown to be mediated by a mixture of M_2_R and M_3_R subtypes [20]. It is well known that the M_3_R stimulates IP_3_-dependent intracellular Ca^2+^-release ([29,2]. However, like S1P [30], stimulation of M_3_R can also activate RhoA-dependent signalling pathways leading to increased myofilament sensitivity of smooth muscle [31,32]. On the other hand, activation of M_2_Rs in airway smooth muscle has been shown to increase the sensitivity of myofilaments to Ca^2+ ^and to inhibit noradrenaline-induced increases in intracellular cAMP [33,34]. In HEK-293 cells, DLS and DMS markedly reduced both M2R-and M3R-mediated increases in [Ca^2+^]_i _[5].

In the present study, we therefore also examined which of these two receptor subtypes (M_2_R or M_3_R) is involved in the SPHK1-dependent constriction of peripheral airways. To address this issue, we carried out a series of functional experiments using PCLS preparations from M_2_R-and M_3_R-deficient mice (M_2_R-KO, M_3_R-KO) and their corresponding wild-type controls (M_2_R-wt, M_3_R-wt). To rule out potential differences in SPHK1 expression between wt and KO animals we initially performed a set of quantitative RT-PCR studies. These studies showed that inactivation of the M_2_R and M_3_R genes had no significant effect on the relative expression levels of SPHK1 compared to the corresponding wt controls. Moreover, confocal laser scanning microscopic studies showed that the localization and distribution of SPHK1 protein were similar in all mouse strains. In agreement with the results of a previous study [20], the peripheral airways of M_3_R-KO mice showed an about 50 % reduction in the magnitude of the initial muscarine induced constriction response, as compared to the corresponding response obtained with preparations from M_3_R-wt mice. We previously demonstrated that the bronchoconstrictor response remaining in the M_3_R-KO mice is exclusively mediated by M_2_Rs [20].

Changes in [Ca^2+^]_i _are the main trigger in the initiation of MR-mediated bronchoconstriction. The initial phase of constriction of intraparenchymal airways is known to be mediated partly via release of Ca^2+ ^from intracellular stores, as shown by the presence of this phase under blockade of ion influx by La^3+ ^or SKF96365. The considerable reduction of the initial constriction by La^3+ ^or SKF96365 further indicates that influx of Ca^2+ ^is also part of this phase of muscarine-mediated constriction. We defined the bronchoconstriction in response to muscarine as 100 % and analyzed the inhibition under various experimental conditions. Blockade of SPHK1 by either DHS or DMS almost completely abolished the initial phase of constriction in bronchi from M_3_R-KO mice (Fig. 5 Tab. 2). In contrast, DHS or DMS only partially reduced this early response in bronchi from M_2_R-KO and wt-mice (Fig. 5). This observation strongly suggests that stimulation of M_2_Rs mediates activation of SPHK1, which eventually triggers the release of Ca^2+ ^from intracellular stores leading to bronchoconstriction. The role of M_2_R in airway smooth muscle contraction appears multi-functional in that the receptor can modulate the function of smooth muscle by activation of multiple signalling pathways including tyrosine kinase activation and stimulation/inhibition of ion channels [35,32]. Phosphorylation of myosin light chain and activation of PKC play important roles in the maintenance of smooth muscle contractions [36,37]. Since S1P has been shown to stimulate myosin phosphorylation [8,38], it is likely that this response is also mediated by the M_2_R subtype.

Our findings strongly suggest that SPHK1 activation is part of the signalling response to M_2_R stimulation in peripheral mouse airways. These airways resemble in their structure and composition of cell types human distal airways [39]. Like human distal airways, murine peripheral airways about 200 μm in diameter that are terminal bronchioles lack submucosal glands, cartilage and contain smooth muscle and Clara cells, in addition to ciliated cells [39]. In studies using monkey and rat peripheral airways, the ACh analogue methacholine (MCh) induced similar responses in large and small mammals [40]. It is therefore likely that the distal airways of man and mice, both of which are also MCh-sensitive [17], share similar MR signalling pathways. In addition, it has been shown that human airway smooth muscle cells constrict in response to S1P [7,8], suggesting that the MR-SPHK1-S1P signalling pathway is also present in human peripheral airways.

## Conclusion

In conclusion, in this study we demonstrated the existence of a novel signalling pathway in the regulation of peripheral airways. We found that MR-mediated constriction of murine peripheral airways is mediated, in part, by activation of SPHK. Our data suggest that muscarinic activation of SPHK contributes to the initial and sustained phase of constriction. The SPHK activation in the initial phase is mainly mediated via the M_2_R subtype. These findings could be of relevance for the development of novel drugs useful for the treatment of chronic obstructive pulmonary disease and asthma. For example, one may speculate that altered S1P signalling may contribute to the hyperreactivity of peripheral airways under pathological conditions.

## List of abbreviations

ACh acetylcholine

CT threshold cycle

MR muscarinic acetylcholine receptor

wt wild-type control

M_2_R-KO muscarinic acetylcholine receptor 2-deficient mice

M_3_R-KO muscarinic acetylcholine receptor 3-deficient mice

PLCβ phospholipase C beta

PCLS Precision cut lung slices

SKF96365 1-2-(4-methoxyphenyl)-2-[3-(4-methoxyphenyl) propoxy]-ethyl-1H-imidazole

S1P sphingosine 1-phosphate

SPHK sphingosine kinases

IP_3 _inositol 1, 4, 5-trisphosphate

DHS D, L-threo-dihydrosphingosine

DMS N, N-dimethyl-sphingosine

NAcS N-acetylsphingosine

MCh Methacholine

## Authors' contributions

PM, NP, SA, WS, SW and RVH carried out the videomorphometric experiments and performed qRT-PCR and immunohistochemistry. JW developed the KO-mice and participated together with WK and RVH in writing and preparation of the manuscript and in the statistical analysis. YB was involved in the design of the study and the immunohistochemical investigations. The data presented in the manuscript are part of the doctoral thesis of PM, NP, SA, WS.
